# Supplementary material for: COZOID: contact zone identifier for visual analysis of protein-protein interactions
Source: BMC Bioinformatics. 2018 Apr 6;19:125. doi: 10.1186/s12859-018-2113-6 (PMC5889581; doi:10.1186/s12859-018-2113-6)
Supplement: Supplementary file 2 — Software build. Executable binary file of the software tool. (ZIP 118,784 kb) [file 12859_2018_2113_MOESM2_ESM.zip › cozoid/java/docs/beansbinding-1.2.1-doc.zip!/org/jdesktop/beansbinding/package-tree.html]

org.jdesktop.beansbinding Class Hierarchy (Beans Binding)


---


|  |  |  |
| --- | --- | --- |
| **Overview** \| **Package** \| Class \| Use \| **Tree** \| **Deprecated** \| **Index** \| **Help** | |  |
| PREV   **NEXT** | **FRAMES**    **NO FRAMES**     **All Classes** |  |


---


## Hierarchy For Package org.jdesktop.beansbinding

**Package Hierarchies:**: All Packages

---

## Class Hierarchy

- java.lang.Object
  - org.jdesktop.beansbinding.**AbstractBindingListener** (implements org.jdesktop.beansbinding.BindingListener)- org.jdesktop.beansbinding.**Binding**<SS,SV,TS,TV>
      - org.jdesktop.beansbinding.**AutoBinding**<SS,SV,TS,TV>
      - org.jdesktop.beansbinding.**Binding.SyncFailure**- org.jdesktop.beansbinding.**Binding.ValueResult**<V>- org.jdesktop.beansbinding.**BindingGroup**- org.jdesktop.beansbinding.**Bindings**- org.jdesktop.beansbinding.**Converter**<S,T>- java.util.EventObject (implements java.io.Serializable)
                  - org.jdesktop.beansbinding.**PropertyStateEvent**
                  - org.jdesktop.beansbinding.**Property**<S,V>
                    - org.jdesktop.beansbinding.**ObjectProperty**<S>- org.jdesktop.beansbinding.**PropertyHelper**<S,V>
                        - org.jdesktop.beansbinding.**BeanProperty**<S,V>- org.jdesktop.beansbinding.**ELProperty**<S,V>
                    - java.lang.Throwable (implements java.io.Serializable)
                      - java.lang.Exception
                        - java.lang.RuntimeException
                          - org.jdesktop.beansbinding.**PropertyResolutionException**
                      - org.jdesktop.beansbinding.**Validator**<T>- org.jdesktop.beansbinding.**Validator.Result**

## Interface Hierarchy

- java.util.EventListener
  - org.jdesktop.beansbinding.**BindingListener**- org.jdesktop.beansbinding.**PropertyStateListener**

## Enum Hierarchy

- java.lang.Object
  - java.lang.Enum<E> (implements java.lang.Comparable<T>, java.io.Serializable)
    - org.jdesktop.beansbinding.**Binding.SyncFailureType**- org.jdesktop.beansbinding.**AutoBinding.UpdateStrategy**

---


|  |  |  |
| --- | --- | --- |
| **Overview** \| **Package** \| Class \| Use \| **Tree** \| **Deprecated** \| **Index** \| **Help** | |  |
| PREV   **NEXT** | **FRAMES**    **NO FRAMES**     **All Classes** |  |


---
